# An indirect comparison of acalabrutinib with and without obinutuzumab vs zanubrutinib in treatment-naive CLL^∗^^†^

**DOI:** 10.1182/bloodadvances.2023012142

**Published:** 2024-04-12

**Authors:** Adam S. Kittai, John N. Allan, Dan James, Helen Bridge, Miguel Miranda, Alan S. M. Yong, Fady Fam, Jack Roos, Vikram Shetty, Alan Skarbnik, Matthew S. Davids

**Affiliations:** 1 Division of Hematology, The Ohio State University Comprehensive Cancer Center, Columbus, OH; 2 Weill Cornell Medicine, New York, NY; 3 Polaris Biostatistics Ltd, Edinburgh, United Kingdom; 4 AstraZeneca, Cambridge Biomedical Campus, Cambridge, United Kingdom; 5 AstraZeneca, Gaithersburg, MD; 6 Novant Health Cancer Institute, Lymphoma and CLL Program, Charlotte, NC; 7 Department of Medical Oncology, Dana-Farber Cancer Institute, Boston, MA

## Abstract

The efficacy and safety of acalabrutinib plus obinutuzumab and acalabrutinib monotherapy vs zanubrutinib in patients with treatment-naive chronic lymphocytic leukemia/small lymphocytic lymphoma without del(17p) were compared using an unanchored matching-adjusted indirect comparison. Individual patient-level data from ELEVATE-TN (acalabrutinib plus obinutuzumab, n = 162; acalabrutinib monotherapy, n = 163) were weighted to match published aggregate baseline data from SEQUOIA cohort 1, which excluded patients with del(17p) (zanubrutinib, n = 241), using variables that were prognostic/predictive of investigator-assessed progression-free survival (INV-PFS) in an exploratory Cox regression analysis of ELEVATE-TN. After matching, INV-PFS was longer with acalabrutinib plus obinutuzumab (hazard ratio [HR], 0.41; 95% confidence interval [CI], 0.23-0.74) and comparable with acalabrutinib monotherapy (HR, 0.91; 95% CI, 0.53-1.56) vs zanubrutinib. Acalabrutinib monotherapy had significantly lower odds of any grade hypertension vs zanubrutinib (odds ratio [OR], 0.44; 95% CI, 0.20-0.99), whereas acalabrutinib plus obinutuzumab had significantly higher odds of neutropenia (OR, 2.19; 95% CI, 1.33-3.60) and arthralgia (OR, 2.33; 95% CI, 1.37-3.96) vs zanubrutinib. No other significant differences in safety were observed. In summary, acalabrutinib plus obinutuzumab had longer INV-PFS with increased odds of neutropenia and arthralgia than zanubrutinib, whereas acalabrutinib monotherapy had similar INV-PFS with lower odds of any grade hypertension. These trials were registered at www.ClinicalTrials.gov as #NCT02475681 and #NCT03336333.

## Key Points

• Acalabrutinib plus obinutuzumab had longer INV-PFS than zanubrutinib, whereas acalabrutinib monotherapy showed no difference.
• The odds of having hypertension were significantly lower with acalabrutinib monotherapy vs zanubrutinib.

## Visual Abstract

**Figure:**
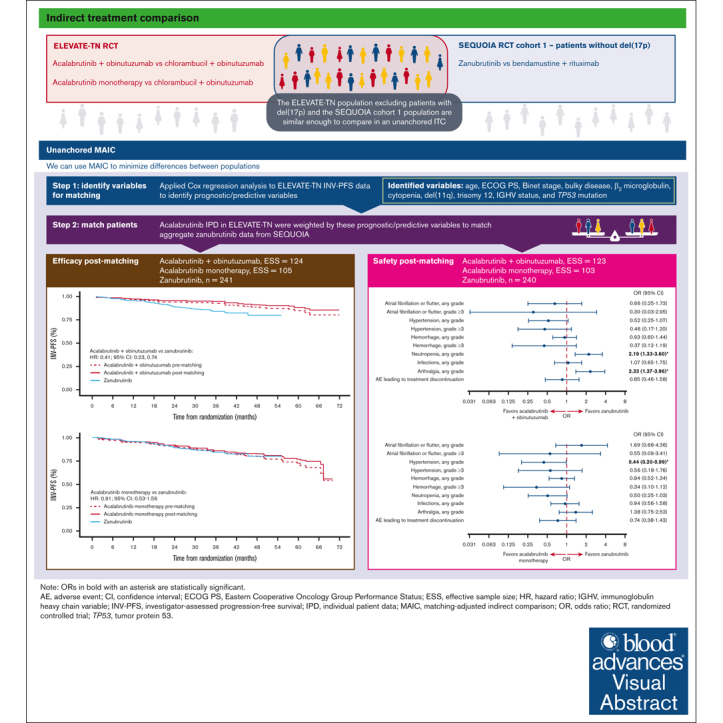

## Introduction

The first-generation Bruton tyrosine kinase inhibitor (BTKi), ibrutinib, is associated with significant adverse events (AEs), such as increased risk of atrial fibrillation (AF), hypertension, and hemorrhage.^1^, ^2^, ^3^, ^4^, ^5^ The second-generation BTKis, acalabrutinib and zanubrutinib, were developed to have a more selective kinase inhibition profile than ibrutinib and, therefore, were predicted to have fewer off-target effects and better safety profiles than ibrutinib.^6^, ^7^, ^8^, ^9^ In the ELEVATE-TN randomized controlled trial (RCT), acalabrutinib plus obinutuzumab and acalabrutinib monotherapy demonstrated superior progression-free survival (PFS) vs chlorambucil plus obinutuzumab over 5 years of follow-up in patients with treatment-naive chronic lymphocytic leukemia (CLL).^10^ Similarly, zanubrutinib demonstrated superior PFS to bendamustine plus rituximab over 3.7 years of follow-up in patients with treatment-naive CLL or small lymphocytic lymphoma (SLL) in cohort 1 of the SEQUOIA RCT, which excluded patients with the del(17p) genetic abnormality.^11^ However, acalabrutinib and zanubrutinib have not been evaluated against each other in a head-to-head RCT in CLL/SLL in the treatment-naive or relapsed/refractory (R/R) setting, and it is unlikely that these trials will be conducted. Consequently, how the 2 second-generation BTKis compare in terms of efficacy and safety in CLL/SLL is unknown, so it is of particular interest to evaluate how these treatments compare when used as first-line treatments for patients with CLL/SLL.

In the absence of head-to-head RCTs, anchored or unanchored indirect treatment comparison (ITC) methods can be used to compare therapeutic arms.^12^ Anchored ITCs require RCTs to have at least 1 treatment arm in common and more complex networks of multiple RCTs with common treatment arms can be used to make pairwise treatment comparisons known as a network meta-analysis.^13^^,^^14^ ELEVATE-TN and SEQUOIA do not share a common treatment arm, meaning an anchored ITC is not feasible. Because of the significant heterogeneity in the RCTs that would be required to connect ELEVATE-TN and SEQUOIA in a network, an unanchored ITC using matching-adjusted indirect comparison (MAIC) was deemed more appropriate than a network meta-analysis. This allows for evaluation of the absolute outcomes of treatments in a nonrandomized crosstrial comparison.^12^^,^^15^ To minimize crosstrial heterogeneity and potential selection bias caused by differences in patient characteristics between studies, MAIC assigns weights to the trial population with available individual patient-level data (IPD) so that it matches the aggregated baseline data of another trial.^12^^,^^16^

An unanchored MAIC was conducted to compare the efficacy and safety of acalabrutinib plus obinutuzumab and acalabrutinib monotherapy vs zanubrutinib monotherapy in patients with treatment-naive CLL/SLL without del(17p) using IPD from ELEVATE-TN^17^ and published aggregate data from cohort 1 of SEQUOIA, which excluded patients with del(17p).^11^^,^^18^

## Methods

This study follows the National Institute for Health and Care Excellence (NICE) guidance on MAIC methodology (Decision Support Unit Technical Support Document 18).^12^

### Matching variables

Matching variables were identified via an exploratory Cox regression analysis of investigator-assessed PFS (INV-PFS) using ELEVATE-TN data and backward stepwise selection. Data from patients without del(17p) from all 3 arms of ELEVATE-TN were combined and evaluated using 1 single model.

In the Cox regression analysis, variables were evaluated to estimate whether they were prognostic (ie, they affected the outcome). Variables were also included as interaction variables with randomized treatment (ie, each variable was multiplied by randomized treatment) to estimate whether they were predictive (ie, they altered the effect of treatment). A 20% significance level was used to identify an inclusive list of factors for matching.

Following guidelines, all prognostic and predictive factors identified that were available for both studies were matched, regardless of whether they were balanced across studies.

The variables reported in both ELEVATE-TN and SEQUOIA that were considered in the matching were age, sex, race, geographical region, time from diagnosis, beta-2 microglobulin, Binet stage, Eastern Cooperative Oncology Group (ECOG) performance status, bulky disease, cytopenia, del(11q), del(13q), trisomy 12, no fluorescence in situ hybridization abnormalities, unmutated immunoglobulin heavy chain variable gene, and *TP53* mutation. Patients with missing Binet data were categorized according to their Rai stage, with a Rai stage less than III equivalent to a Binet stage of A or B.

Using the matching variables, weights were estimated for the acalabrutinib plus obinutuzumab and acalabrutinib monotherapy groups. The weights were then rescaled to sum up to the sample size of the acalabrutinib plus obinutuzumab and acalabrutinib monotherapy arms, with a rescaled weight >1 indicating that an individual in ELEVATE-TN carried more weight than they carried before weighting in the original data set. The estimated weights were reported using summary statistics and histogram plots. These were inspected to identify extreme weights (eg, >10), which would indicate that a MAIC was excessively influenced by a small number of patients. The effective sample size (ESS) was calculated to approximate the sample size that would be required to obtain a similar level of precision as the weighted data and to assess how much information was lost in the matching.

The baseline characteristics of the acalabrutinib plus obinutuzumab and acalabrutinib monotherapy arms before and after matching were compared with the published baseline data for zanubrutinib to check whether matching minimized differences.

### Efficacy analysis

The analysis set for acalabrutinib plus obinutuzumab and acalabrutinib monotherapy in the efficacy MAIC comprised randomized patients without del(17p) in ELEVATE-TN at the October 2021 data cutoff (DCO; acalabrutinib plus obinutuzumab, n = 162; acalabrutinib monotherapy, n = 163; median follow-up, 58 months). The analysis set for zanubrutinib comprised the intention-to-treat population of cohort 1 without del(17p) from SEQUOIA at the October 2022 DCO (n = 241; median follow-up, 44 months).^18^

INV-PFS was evaluated because this was the most mature PFS end point reported in both ELEVATE-TN and SEQUOIA. In ELEVATE-TN, independent review committee–assessed PFS (IRC-PFS) was collected up to the primary analysis (DCO February 2019; median follow-up, 28 months), after which only INV-PFS was assessed. In SEQUOIA, IRC-PFS was only reported at the May 2021 DCO (median follow-up, 26 months). The data available for IRC-PFS (supplementary Table 1) are therefore far less mature than those available for INV-PFS in both ELEVATE-TN and SEQUOIA. Using the most mature data enables greater precision in the estimation of treatment effect than using immature data with few IRC-PFS events.

The individual event times and event states (ie, whether the patient experienced an event or was censored) for zanubrutinib were digitally extracted from the Kaplan-Meier plots reporting INV-PFS in SEQUOIA using the algorithm by Guyot et al.^19^ These data were combined with data on the number of events and number of patients at risk over time to generate pseudo-IPD, which were then combined with the weighted efficacy IPD for acalabrutinib plus obinutuzumab and acalabrutinib monotherapy. The hazard ratios (HRs) for INV-PFS comparing acalabrutinib plus obinutuzumab and acalabrutinib monotherapy with zanubrutinib were generated using weighted Cox regression models fitted to each combined data set. The 95% confidence intervals (CIs) were estimated using a robust sandwich estimator of the standard errors. This accounted for the weights being estimated rather than fixed and known.

To assess how matching affected outcomes, Kaplan-Meier plots of INV-PFS for acalabrutinib plus obinutuzumab and acalabrutinib monotherapy before and after matching were generated and compared. To compare acalabrutinib plus obinutuzumab and acalabrutinib monotherapy with zanubrutinib, Kaplan-Meier estimates of 36-month INV-PFS were calculated.

#### Efficacy sensitivity analysis

A sensitivity analysis was conducted to assess whether the efficacy results from the primary analysis remained consistent after adding all possible variables that could be used for matching, regardless of whether they were found to be predictive or prognostic of INV-PFS using ELEVATE-TN data.

### Safety analysis

The safety analysis assessed the incidence of AEs and reported the odds ratios (ORs) of AEs occurring with acalabrutinib plus obinutuzumab and acalabrutinib monotherapy vs zanubrutinib. The incidence of AEs is time sensitive and cumulative; therefore, the safety analysis was conducted using the September 2020 DCO from ELEVATE-TN, which ensured that the median follow-up for acalabrutinib plus obinutuzumab and acalabrutinib monotherapy (both 47 months) was comparable with the median follow-up for zanubrutinib in SEQUOIA at the October 2022 DCO (44 months).^18^ Median drug exposure was not reported at this SEQUOIA DCO; therefore, median follow-up was compared instead.

The safety analysis set for acalabrutinib plus obinutuzumab and acalabrutinib monotherapy comprised any patient without del(17p) who had received the study drug in ELEVATE-TN (safety set included acalabrutinib plus obinutuzumab, n = 162; and acalabrutinib monotherapy, n = 162). The analysis set for zanubrutinib comprised the safety analysis population of cohort 1 in SEQUOIA (n = 240).^18^ The acalabrutinib plus obinutuzumab and acalabrutinib monotherapy populations were matched to the intention-to-treat population for zanubrutinib because aggregate baseline data had not been published for the zanubrutinib safety population. The matching variables used for the safety analysis were the same as those used for the efficacy analysis. AEs of interest that were common to acalabrutinib and zanubrutinib and were reported in both trials were evaluated.

To assess how matching affected the results, a prematched analysis was performed in which the ORs of AEs with acalabrutinib plus obinutuzumab and acalabrutinib monotherapy vs zanubrutinib were estimated via logistic regression fitted to the safety end points of interest and supported by reported frequencies of each AE category by treatment arm. For the matched results, pseudo-IPD for SEQUOIA were created using the number of patients with and without the AEs of interest reported.^18^ These data were combined with the patient-level safety data from ELEVATE-TN. Weighted logistic regression analysis was performed to correct for between-trial imbalances in baseline characteristics. The postmatching ORs were reported with 95% CIs that were calculated using robust standard errors to account for the uncertainty introduced by the matching.

#### Safety sensitivity analysis

A safety sensitivity analysis was conducted, in which matching was based on characteristics considered relevant for safety by clinical experts. These were age, ECOG performance status, and cytopenia.

### Analysis of statistical significance

For both the efficacy and safety MAICs, statistical significance was set at the 5% level. No tests were prespecified, and no correction was made for multiple testing.

## Results

### Matching variables

Variables that were identified to be prognostic or predictive of INV-PFS in the Cox regression analysis conducted using ELEVATE-TN data were age, beta-2 microglobulin, Binet stage, ECOG Performance Status, bulky disease, cytopenia, del(11q), trisomy 12, unmutated immunoglobulin heavy chain variable gene, and *TP53* status (supplemental Table 2). Categorical age (<65 vs ≥65 years) was not evaluated in the Cox regression analysis but was included in the matching, because there were imbalances in categorical age between the treatment groups when patients were matched based on continuous age but not categorical age.

The median scaled weights for acalabrutinib plus obinutuzumab and acalabrutinib monotherapy were 0.85 (range, 0.28-3.06) and 0.73 (range, 0.19-4.29), respectively, and there were no excessive weights (>10; supplemental Figure 1), which indicated that none of the patients had an excessive influence on outcomes. After matching, the ESSs of the acalabrutinib plus obinutuzumab and acalabrutinib monotherapy arms were 124 and 105, respectively (77% and 64% of the original efficacy samples, respectively). Baseline characteristics before and after matching are reported in Table 1.

**Table 1.** **Baseline characteristics of the acalabrutinib plus obinutuzumab and acalabrutinib monotherapy arms in ELEVATE-TN vs the zanubrutinib arm in SEQUOIA**

Baseline variable, %	Acalabrutinib plus obinutuzumab before matching (N = 162)	Acalabrutinib plus obinutuzumab after matching, (ESS = 124)∗	Acalabrutinib monotherapy before matching (N = 163)	Acalabrutinib monotherapy after matching, (ESS = 105)†	Zanubrutinib (N = 241)
**Age**					
Median, y	70.0	69.6	70.0	69.6	70.0
<65 y	18.5	18.7	14.1	18.7	18.7
Sex, female	34.6	34.5	36.2	38.9	36.1
Race, White	91.4	92.6	95.1	92.8	91.7
Region, Europe	55.6	53.3	50.3	43.3	72.2
Median time from initial diagnosis, mo	30.6	31.8	25.4	30.4	31.3
Cancer type, CLL	100.0	100.0	100.0	100.0	91.7†
Beta-2 microglobulin, >3.5 mg/L	74.1	56.0	79.1	56.0	56.0
Binet stage, A/B	56.2	71.0	58.9	71.0	71.0
ECOG PS, 0-1	95.7	93.8	92.6	93.8	93.8
Bulky disease, ≥5 cm	25.9	28.6	38.7	28.6	28.6
Cytopenia at baseline	51.9	42.3	47.2	42.3	42.3
**Mutation status**					
Del(11q)	18.5	17.8	18.4	17.8	17.8
Del(13q)	53.7	58.4	61.3	70.1	56.4
Trisomy 12	24.7	18.7	29.4	18.7	18.7
FISH abnormalities, absent	24.1	24.6	11.0	11.3	23.2
IGHV, unmutated	58.0	51.9	65.6	51.9	51.9
*TP53*, mutated	4.9	6.2	4.3	6.2	6.2
Complex karyotype‡	12.3	13.5	13.5	14.5	NR

Data are reported as percentages (%), unless otherwise specified.

FISH, fluorescent in situ hybridization; IGHV, immunoglobulin heavy chain variable gene; NR, not reported; PS, performance status; *TP53*, tumor protein 53.

∗ The number of patients was calculated using the rescaled weights and therefore sum to the efficacy analysis sample size for acalabrutinib plus obinutuzumab (n = 162) and acalabrutinib monotherapy (n = 163).

† The remaining 8.3% of patients had SLL.

‡ Complex karyotype was defined as ≥3 cytogenetic abnormalities based on karyotyping by the central laboratory.

### Efficacy analysis

**Figure 1.**
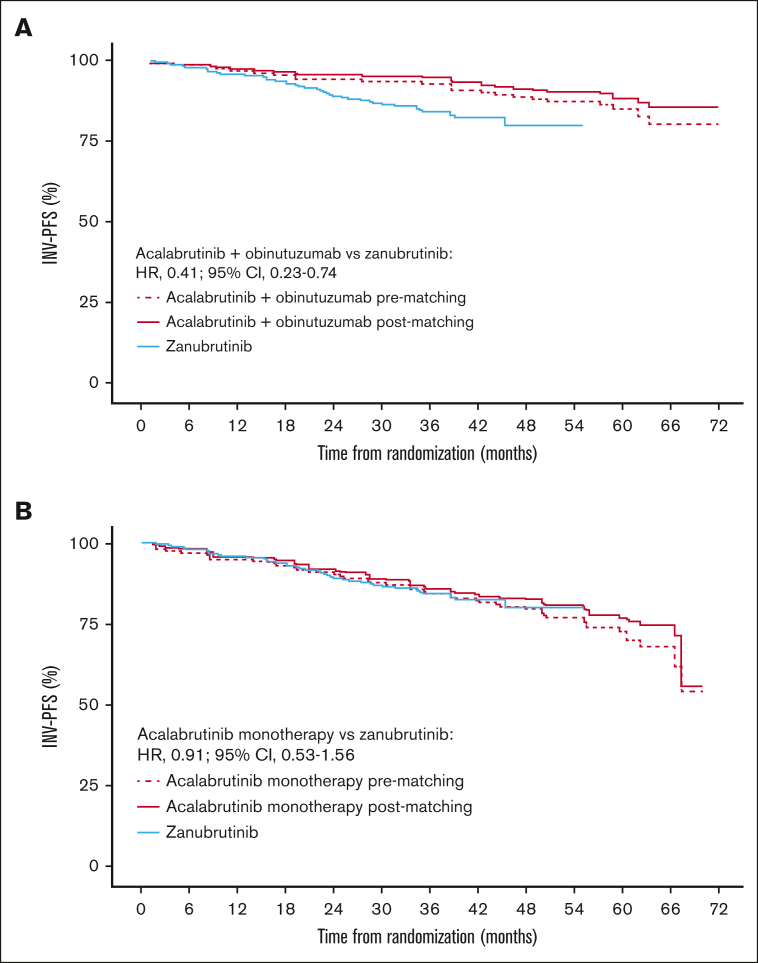
**Efficacy results.** Kaplan-Meier plot of INV-PFS for acalabrutinib plus obinutuzumab (A) and acalabrutinib monotherapy (B) before and after matching vs zanubrutinib.

Matching patient characteristics across the studies led to only small changes in INV-PFS with acalabrutinib plus obinutuzumab and acalabrutinib monotherapy vs zanubrutinib (Figure 1). There was also no difference between IRC-PFS and INV-PFS before matching, as shown in supplemental Table 1.

#### Acalabrutinib plus obinutuzumab vs zanubrutinib

After matching, acalabrutinib plus obinutuzumab had a higher 36-month INV-PFS (95%; 95% CI, 90-97) than zanubrutinib (84%; 95% CI, 79-88). The MAIC-weighted Cox HR showed INV-PFS to be longer with acalabrutinib plus obinutuzumab vs zanubrutinib (HR, 0.41; 95% CI, 0.23-0.74; Figure 1A).

#### Acalabrutinib monotherapy vs zanubrutinib

Acalabrutinib monotherapy after matching had a similar 36-month INV-PFS (86%; 95% CI, 78-91) to zanubrutinib (84%; 95% CI, 79-88), and the MAIC-weighted Cox HR indicated that there was no significant difference vs zanubrutinib (HR, 0.91; 95% CI, 0.53-1.56; Figure 1B).

#### Efficacy sensitivity analysis

The efficacy sensitivity analysis assessed the impact of including all possible variables in the matching, regardless of whether they were found to be prognostic or predictive of INV-PFS. The median weights for acalabrutinib plus obinutuzumab and acalabrutinib monotherapy were close to 1, and there were no excessive weights (>10; supplemental Figure 2). After matching, the ESSs of the acalabrutinib plus obinutuzumab and acalabrutinib monotherapy arms were 103 and 67, respectively (63% and 41% of the original efficacy samples, respectively).

The results after matching were consistent with the main analysis. INV-PFS was longer with acalabrutinib plus obinutuzumab vs zanubrutinib (HR, 0.41; 95% CI, 0.21-0.77), and there was no significant difference between acalabrutinib monotherapy and zanubrutinib (HR, 0.84; 95% CI, 0.44-1.58).

### Safety analysis

**Figure 2.**
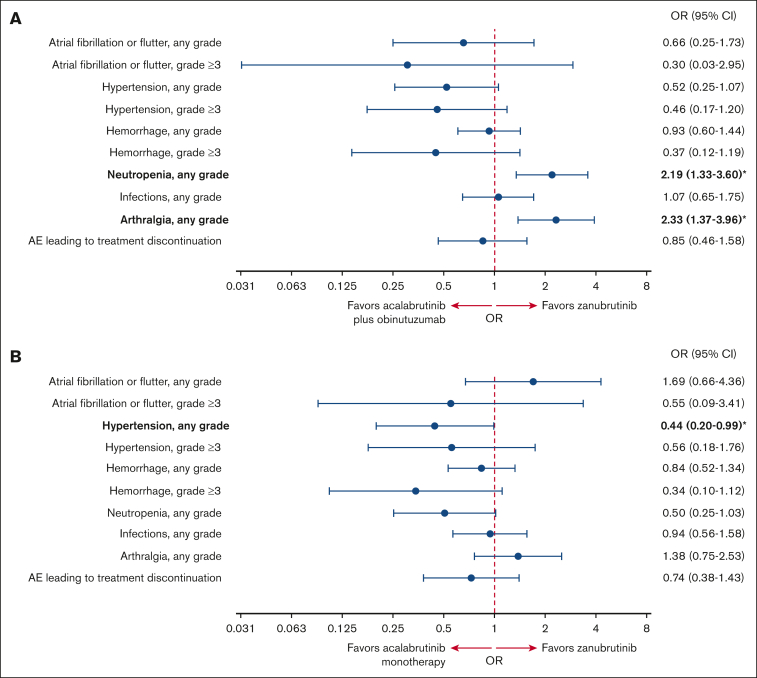
**Safety analysis.** Forest plot showing the OR of AEs after matching with (A) acalabrutinib plus obinutuzumab and (B) acalabrutinib monotherapy vs zanubrutinib. ORs in bold with an asterisk are statistically significant.

In the safety analysis, the acalabrutinib plus obinutuzumab and acalabrutinib monotherapy ESSs after matching were 123 and 103, respectively (76% and 64% of the original safety samples, respectively; supplemental Figure 3). The incidence of AEs before and after matching is reported in supplemental Table 3. The ORs of different safety outcomes after matching are shown in Figure 2. Some AEs occurred in few patients, such as grade ≥3 atrial fibrillation (AF)/atrial flutter, grade ≥3 hemorrhage, and grade ≥3 hypertension. Consequently, the CIs for these ORs were very wide. In addition, some of the results in either direction were only marginally significant or nonsignificant.

#### Acalabrutinib plus obinutuzumab vs zanubrutinib

For cardiovascular AEs, there was no evidence of a difference in the odds of having AF/atrial flutter (any grade OR, 0.66; 95% CI, 0.25-1.73; grade ≥3 OR, 0.30; 95% CI, 0.03-2.95), hemorrhage (any grade OR, 0.93; 95% CI, 0.60-1.44; grade ≥3 OR, 0.37; 95% CI, 0.12-1.19), or hypertension (any grade OR, 0.52; 95% CI, 0.25-1.07; grade ≥3 OR, 0.46; 95% CI, 0.17-1.20) with acalabrutinib plus obinutuzumab vs zanubrutinib.

When looking at other safety outcomes, the odds of having any grade neutropenia (OR, 2.19; 95% CI, 1.33-3.60) and arthralgia (OR, 2.33; 95% CI, 1.37-3.96) were significantly higher with acalabrutinib plus obinutuzumab vs zanubrutinib. There was no evidence of a difference in the odds of having any grade infections (OR, 1.07; 95% CI, 0.65-1.75) or AEs leading to treatment discontinuation (OR, 0.85; 95% CI, 0.46-1.58) with acalabrutinib plus obinutuzumab vs zanubrutinib.

#### Acalabrutinib monotherapy vs zanubrutinib

For cardiovascular AEs, the odds of having any grade hypertension were significantly lower with acalabrutinib monotherapy vs zanubrutinib (OR, 0.44; 95% CI, 0.20-0.99). There was no evidence of a difference in the odds of having AF/atrial flutter (OR, 1.69; 95% CI, 0.66-4.36; grade ≥3 OR, 0.55; 95% CI, 0.09-3.41), hemorrhage (any grade OR, 0.84; 95% CI, 0.52-1.34; grade ≥3 OR, 0.34; 95% CI, 0.10-1.12), or grade ≥3 hypertension (OR, 0.56; 95% CI, 0.18-1.76) with acalabrutinib monotherapy vs zanubrutinib.

With regard to other safety outcomes, there was no evidence of a difference between acalabrutinib monotherapy and zanubrutinib in the odds of having any grade neutropenia (OR, 0.50; 95% CI, 0.25-1.03), arthralgia (OR, 1.38; 95% CI, 0.75-2.53), infections (OR, 0.94; 95% CI, 0.56-1.58), or AEs leading to treatment discontinuation (OR, 0.74; 95% CI, 0.38-1.43).

#### Safety sensitivity analysis

The safety sensitivity analysis assessed the impact of only including variables that were specifically thought to influence safety outcomes in the matching (age, ECOG performance status, and cytopenia). The median weights for acalabrutinib plus obinutuzumab and acalabrutinib monotherapy were close to 1, with no weights <0.7 or >2.0 (supplemental Figure 4). After matching, the ESSs of the acalabrutinib plus obinutuzumab and acalabrutinib monotherapy arms were 154 and 157, respectively (95% and 97% of the original safety samples, respectively).

The results of the sensitivity analysis (supplemental Figure 5) were generally consistent with those of the main analysis; however, there were a few differences at the 5% statistical significance level. The odds of having any grade hypertension were significantly lower with acalabrutinib plus obinutuzumab vs zanubrutinib (OR, 0.47; 95% CI, 0.24-0.92), and the odds of having grade ≥3 hypertension (OR, 0.31; 95% CI, 0.11-0.85) were significantly lower with acalabrutinib monotherapy than with zanubrutinib.

## Discussion

This MAIC estimates the comparative efficacy and safety of the 2 second-generation BTKis, acalabrutinib (in combination with obinutuzumab and as a monotherapy) and zanubrutinib, in patients with treatment-naive CLL/SLL without del(17p). This analysis showed that the efficacy of acalabrutinib plus obinutuzumab was improved compared with zanubrutinib in terms of INV-PFS, whereas there was no evidence of a difference in efficacy between acalabrutinib monotherapy and zanubrutinib.

When looking at safety, our results showed significantly lower odds of having any grade hypertension with acalabrutinib monotherapy vs zanubrutinib. Acalabrutinib plus obinutuzumab had significantly higher odds of any grade neutropenia and arthralgia than zanubrutinib. An increase in the odds of some AEs is expected when comparing a combination regimen with a monotherapy.^20^ For example, higher rates of neutropenia and arthralgia were observed with acalabrutinib plus obinutuzumab vs acalabrutinib monotherapy in the ELEVATE-TN RCT.^17^ Indeed, in this analysis, the odds of any grade neutropenia were significantly higher with acalabrutinib plus obinutuzumab vs zanubrutinib but not with acalabrutinib monotherapy vs zanubrutinib, which indicates that the increase in neutropenia is likely to be caused by obinutuzumab, with other studies also linking obinutuzumab to neutropenia.^21^, ^22^, ^23^ The increased odds of certain AEs, such as neutropenia, should be balanced against the potentially improved efficacy of acalabrutinib plus obinutuzumab vs zanubrutinib when making treatment decisions, as well as patients’ preferences.

The results from this analysis align with those from a similar MAIC conducted in patients with R/R CLL,^24^ which did not find any difference in the efficacy between acalabrutinib monotherapy and zanubrutinib. Both MAICs found that although the safety profiles were largely similar, there were some differences between the 2 BTKis; notably, the odds of having any grade hypertension were significantly lower with acalabrutinib monotherapy vs zanubrutinib in treatment-naive and R/R CLL. In R/R CLL, the odds of having any grade hemorrhage were significantly lower with acalabrutinib monotherapy vs zanubrutinib, whereas there was no evidence of a difference in patients with treatment-naive CLL/SLL. The results of both MAICs found no evidence of a difference in the odds of AF/atrial flutter between acalabrutinib with or without obinutuzumab and zanubrutinib. However, different matching variables were used in the R/R MAIC compared with this analysis.

Despite patients with del(17p) being enrolled in ELEVATE-TN and other cohorts from SEQUOIA, outcomes in this subpopulation could not be evaluated. This was because, although SEQUOIA included a separate large cohort of patients with del(17p) (n = 111), there were too few patients with del(17p) in ELEVATE-TN (acalabrutinib plus obinutuzumab, n = 17; acalabrutinib monotherapy, n = 16).^11^^,^^17^ Therefore, after matching, the ESSs would have been too small to produce meaningful results. Combining and comparing the population with and without del(17p) in SEQUOIA and ELEVATE-TN would not have been possible either because there would have been a large imbalance in the proportion of patients with del(17p), a factor known to affect outcomes.^25^^,^^26^

MAICs have been increasingly applied in a variety of disease areas, including CLL.^27^^,^^28^ In the absence of direct head-to-head evidence, this technique has informed the decision-making of health technology assessment bodies, such as NICE in the United Kingdom. For example, NICE accepted the use of MAIC to compare acalabrutinib with ibrutinib in its evaluation of acalabrutinib in R/R CLL,^29^ before the readout of the ELEVATE-RR RCT.^8^ One of the strengths of our study was that it followed the published NICE guidance on MAIC methodology (Decision Support Unit Technical Support Document 18).^12^^,^^30^ In addition, ELEVATE-TN and SEQUOIA had very similar baseline characteristics, which meant that matching had little impact on the results and led to a small reduction in ESS. Indeed, the reduction in ESS from the overall sample size with acalabrutinib plus obinutuzumab and acalabrutinib monotherapy was 24% and 36%, respectively. This is considerably lower than the median reduction in ESS (74%) reported in a review of ITCs submitted to NICE,^30^ highlighting the good overlap between the ELEVATE-TN and SEQUOIA populations. The changes observed in INV-PFS and in the incidence of AEs with acalabrutinib plus obinutuzumab and acalabrutinib monotherapy before vs after matching were also modest, further confirming the similarity of the populations in these 2 trials.

A large number of variables (10 in total) were identified and used for matching; these were identified to be prognostic/predictive of INV-PFS in the exploratory Cox regression analysis of ELEVATE-TN that was conducted before matching. Most variables were also shown to be prognostic/predictive in published literature.^31^ The efficacy results were robust and were not affected by increasing the number of variables matched on to all possible variables in the sensitivity analysis, indicating that all relevant variables had already been matched on in the primary analysis. However, there was a larger reduction in the ESS in the sensitivity analysis than the primary analysis (acalabrutinib plus obinutuzumab, 37% vs 24%; acalabrutinib monotherapy, 59% vs 36%), leading to a slight loss of precision in the results. For consistency with the efficacy analysis, the same variables were used for the primary safety analysis. However, the variables that affect efficacy outcomes may differ from those that affect safety outcomes; for this reason, the safety sensitivity analysis was conducted using only matching variables considered to specifically affect safety outcomes. Compared with the primary safety analysis, the reductions in the ESS were much smaller in the safety sensitivity analysis (acalabrutinib plus obinutuzumab, 5% vs 24%; acalabrutinib monotherapy, 3% vs 36%). This indicates that, before matching, the ELEVATE-TN and SEQUOIA populations were well balanced with respect to age, ECOG performance status, and cytopenia. Because the ESSs were larger, the sensitivity analysis results had a higher level of precision than the primary safety analysis. Consequently, there were more significant differences in the sensitivity analysis than in the primary analysis. For example, in the sensitivity analysis, there was a significant reduction in the odds of having any grade hypertension with acalabrutinib plus obinutuzumab and grade ≥3 hypertension with acalabrutinib monotherapy.

This study has potential limitations that are inherent to the methodology and specific to this analysis. The unanchored MAIC methodology makes strong and untestable assumptions that all prognostic and predictive variables have been adequately adjusted for, and it is not possible to determine the extent of bias. Despite matching on all observed patient variables available for both studies that were prognostic/predictive at baseline, unobserved variables or variables reported by only 1 study cannot be controlled for via MAIC. For example, SEQUOIA was conducted more recently than ELEVATE-TN and may have been affected by the COVID-19 pandemic, but it was not possible to adjust for the impact of COVID-19 infections on INV-PFS. The difference in time period also meant physicians may have had more knowledge about BTKis and managing the associated AEs during SEQUOIA than during ELEVATE-TN. For example, unlike ELEVATE-TN, SEQUOIA allowed dose reductions to manage AEs, which may have affected the frequency and severity of AEs in SEQUOIA. Patients who relapsed in SEQUOIA may also have had more treatment options.

This study was also limited by the data that were not publicly available for SEQUOIA, particularly regarding baseline variables (which, therefore, could not be included in the matching), AEs, and treatments to manage AEs. Indeed, the incidence of headache could not be evaluated because this was not publicly available for zanubrutinib at the most recent DCO. IPD were not available for SEQUOIA, and it is unclear whether different matching variables would have been selected if a Cox regression analysis had been applied to SEQUOIA data or if SEQUOIA patients had been matched to ELEVATE-TN patients. However, the similarity between populations makes it unlikely that this would have yielded different conclusions. Another limitation of the analysis is that OS was not assessed in this MAIC because the data were not mature, and too few OS events had occurred at the most recent DCOs to generate meaningful results. Finally, the analysis was not prespecified before the ELEVATE-TN and SEQUOIA RCTs were conducted, which affected the reliability of the results, and multiplicity was not adjusted for. This means that multiple 95% CIs were estimated without adjusting for the possibility that significant differences might have been observed by chance or because multiple end points were evaluated.

In summary, this analysis suggests that in patients with treatment-naive CLL/SLL without del(17p), when matching on patient baseline characteristics that were found to be prognostic or predictive of INV-PFS, acalabrutinib plus obinutuzumab may be more efficacious in terms of INV-PFS than zanubrutinib, whereas there was no evidence of a difference in the efficacy of acalabrutinib monotherapy and zanubrutinib. The odds of having any grade hypertension were significantly lower with acalabrutinib monotherapy than with zanubrutinib, whereas the odds of having any grade neutropenia and arthralgia were significantly higher with acalabrutinib plus obinutuzumab than with zanubrutinib. This analysis can help inform clinical decision-making, including the consideration of the risk of AEs when counseling patients; however, this analysis should be considered alongside all other treatment characteristics. Despite the limitations inherent to MAIC analyses, this study provides a valuable systematic comparison of commonly used regimens for which randomized, prospective data are not available and are not expected to be generated.

Conflict-of-interest disclosure: A.S.K. has received consulting fees from AbbVie, AstraZeneca, BeiGene, Janssen, Kite, a Gilead company, and Loxo@Lilly; has ongoing research funding from 10.13039/100004325AstraZeneca and 10.13039/100017239BeiGene; and is part of the speakers bureau for BeiGene. J.N.A. has received consulting fees from AbbVie, ADC Therapeutics, AstraZeneca, BeiGene, Epizyme/Ipsen, Genentech, Janssen, Lava Therapeutics, Lilly, Pharmacyclics, and TG Therapeutics; has received research funding from 10.13039/100017239BeiGene, 10.13039/100006436Celgene, 10.13039/100004328Genentech, and 10.13039/100005565Janssen; and is part of the speakers bureau for AbbVie, BeiGene, and Janssen/Pharmacyclics. D.J. has received consulting fees from AstraZeneca, United Kingdom. H.B., M.M., and F.F. are employees of AstraZeneca, United Kingdom. A.S.M.Y., J.R., and V.S. are employees of AstraZeneca, United States. H.B., M.M., A.S.M.Y., and J.R. own stocks in AstraZeneca. J.R. owns patents/royalties/other intellectual property for CalciMedica. V.S. owns stocks for Verona Pharma. A.S. holds a consulting or advisory role with AbbVie, AstraZeneca, Epizyme, Genentech, Genmab, Janssen, Kite, a Gilead company, Loxo@Lilly, MorphoSys, Novartis, Pharmacyclics, Seagen, and TG Therapeutics, and is a member of the speakers bureau for AbbVie, ADC Therapeutics, AstraZeneca, BeiGene, Celgene, Genentech, Janssen, Jazz Pharmaceuticals, Kite, a Gilead company, Loxo@Lilly, Pharmacyclics, Seagen, and TG Therapeutics. M.S.D. has received institutional research funding from 10.13039/100006483AbbVie, 10.13039/100004325AstraZeneca, 10.13039/100019621Ascentage Pharma, 10.13039/100004328Genentech, 10.13039/100016282MEI Pharma, 10.13039/100004336Novartis, Surface Oncology, and 10.13039/100013252TG Therapeutics, and personal consulting income from AbbVie, Adaptive Biosciences, Ascentage Pharma, AstraZeneca, BeiGene, Bristol Myers Squibb, Eli Lilly, Genentech, Genmab, Janssen, Merck, MingSight Pharmaceuticals, Nuvalent, Secura Bio, TG Therapeutics, and Takeda Pharmaceuticals.
