# Supplementary material for: An indirect comparison of acalabrutinib with and without obinutuzumab vs zanubrutinib in treatment-naive CLL
Source: Blood Adv. 2024 Apr 12;8(11):2861–9. doi: 10.1182/bloodadvances.2023012142 (PMC11176945; doi:10.1182/bloodadvances.2023012142)
Supplement: Supplemental Tables and Figures [file BLOODA_ADV-2023-012142-mmc1.pdf]

An indirect comparison of acalabrutinib with and without obinutuzumab versus zanubrutinib in treatment-naive CLL

Supplementary materials

Supplementary Table 1. Unmatched 24-month IRC-PFS and INV-PFS in ELEVATE-TN at the 8 February 2019 DCO and SEQUOIA at the 7 May 2021 DCO

Study	ELEVATE-TN[17]		SEQUOIA[11]
Treatment arm	Acalabrutinib plus obinutuzumab	Acalabrutinib monotherapy	Zanubrutinib
IRC-PFS, % (95% CI)	93 (87-96)	87 (81-92)	88.9 (81.3-93.6)
INV-PFS, % (95% CI)	92 (87-95)	90 (85-94)	87.0 (79.0-92.1)

CI, confidence interval; DCO, data cut-off; INV, investigator-assessed; IRC, independent review committee; PFS, progression-free survival.

Supplementary Table 2. Variables that were statistically significant in the exploratory Cox regression analyses

Prognostic variables
Beta-2 microglobulin (>3.5 vs ≤3.5 mg/L)
ECOG PS (0-1 vs ≥2)
Bulky disease (yes vs no)
TP53 mutation (yes vs no)
**Prognostic and predictive variables**
Age (years)
Binet stage (A/B vs C)
Cytopenia at baseline (yes vs no)
del(11q) (yes vs no)
Trisomy 12 (yes vs no)
IGHV (unmutated vs mutated)

Note: this table reports the variables that were found to be prognostic/predictive of the outcome at a 20% significance level during backward stepwise selection and that were available for both studies. These variables were included in the matching to weight IPD from ELEVATE-TN to match published baseline characteristics in SEQUOIA. A 20% significance level was chosen to provide an inclusive list of factors for matching.
ECOG PS, Eastern Cooperative Oncology Group Performance Status;
IGHV, immunoglobulin heavy chain variable gene; IPD, individual patient-level data;
TP53, tumor protein 53.

Supplementary Table 3. Incidence of AEs pre- and post-matching

AE, n (%)	Acalabrutinib plus obinutuzumab pre-matching (N = 162)	Acalabrutinib plus obinutuzumab post-matching (ESS = 123)	Acalabrutinib monotherapy pre-matching (N = 162)	Acalabrutinib monotherapy post-matching (ESS = 162)	Zanubrutinib (N = 240)
**Cardiovascular AEs**					
**AF or atrial flutter**					
Any grade	7 (4.3)	4 (3.3)	11 (6.8)	8 (8.2)	12 (5.0)
Grade ≥3	1 (0.6)	0 (0.4)	2 (1.2)	1 (0.7)	3 (1.2)
**Hemorrhage**					
Any grade	73 (45.1)	58 (46.9)	68 (42.0)	45 (44.3)	117 (48.8)
Grade ≥3	4 (2.5)	3 (2.3)	4 (2.5)	2 (2.1)	14 (5.8)
**Hypertension**					
Any grade	13 (8.0)	12 (9.9)	13 (8.0)	9 (8.5)	42 (17.5)
Grade ≥3	6 (3.7)	5 (4.4)	5 (3.1)	5 (5.3)	22 (9.2)
**Other AEs, any grade**					
Arthralgia	44 (27.2)	37 (29.8)	31 (19.1)	21 (20.1)	37 (15.4)
Infections	121 (74.7)	91 (74.2)	120 (74.1)	74 (71.7)	175 (72.9)
Neutropenia	58 (35.8)	38 (30.5)	19 (11.7)	9 (9.2)	40 (16.7)
**Treatment tolerability**					
AE leading to treatment discontinuation	23 (14.2)	16 (13.1)	23 (14.2)	12 (11.5)	36 (15.0)

Data reported are n (%).
AE, adverse event; AF, atrial fibrillation; ESS, effective sample size.

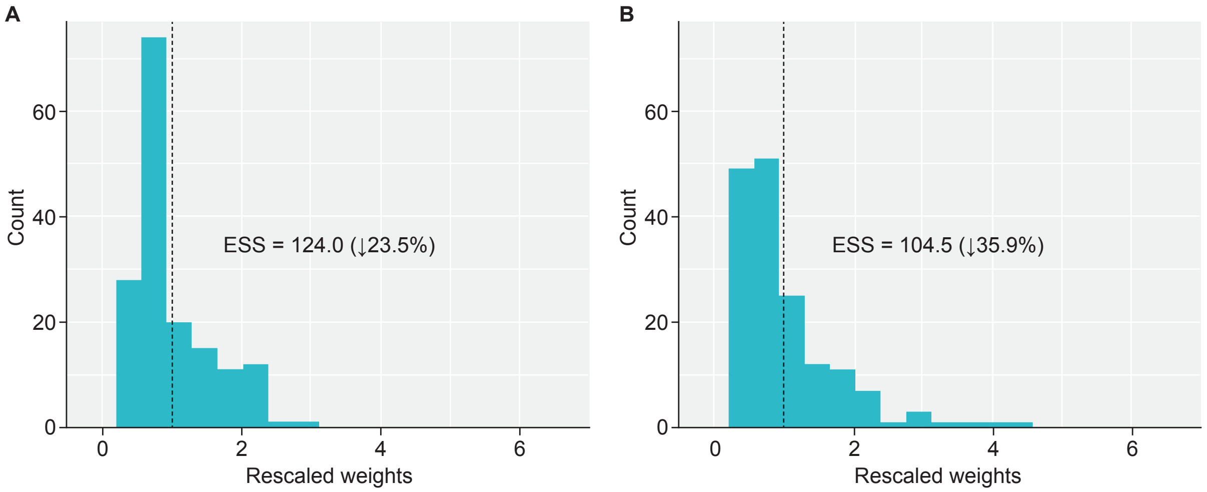

Supplementary Figure 1. Histogram plot of weights in the primary efficacy analysis for (A) acalabrutinib plus obinutuzumab and (B) acalabrutinib monotherapy.

This histogram of the rescaled weights from this MAIC analysis shows that the distribution is mainly concentrated about 1 and there are no extreme weights (>10), which would indicate that a small minority of patients had a disproportionately large influence on outcomes. The reduction in ESS (which in itself is a derivative of the raw weights) was also comparatively small (23.5% for acalabrutinib plus obinutuzumab and 35.9% for acalabrutinib monotherapy). This is an indicator of the degree of overlap in baseline characteristics across the two studies. A large reduction in ESS (>70%) has been shown to increase the level of bias in any indirect treatment comparisons.
ESS, effective sample size; MAIC, matching-adjusted indirect comparison.

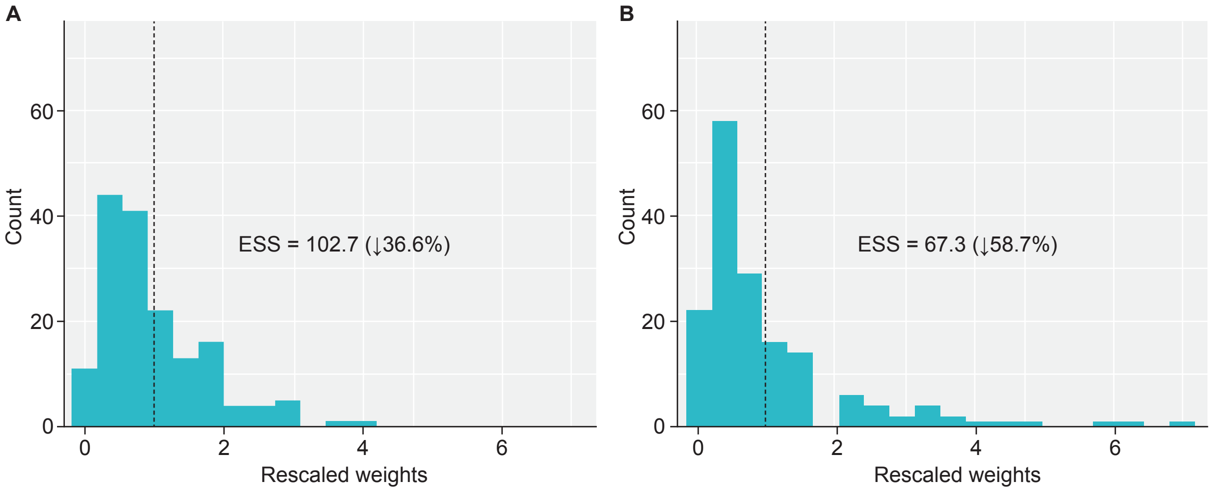

Supplementary Figure 2. Histogram plot of weights in the efficacy sensitivity analysis for (A) acalabrutinib plus obinutuzumab and (B) acalabrutinib monotherapy.

This histogram of the rescaled weights from this MAIC analysis shows that the distribution is mainly concentrated about 1 and there are no extreme weights (>10), which would indicate that a small minority of patients had a disproportionately large influence on outcomes. The reduction in ESS (which in itself is a derivative of the raw weights) was also comparatively small (36.6% for acalabrutinib plus obinutuzumab and 58.7% for acalabrutinib monotherapy). This is an indicator of the degree of overlap in baseline characteristics across the two studies. A large reduction in ESS (>70%) has been shown to increase the level of bias in any indirect treatment comparisons.
ESS, effective sample size; MAIC, matching-adjusted indirect comparison.

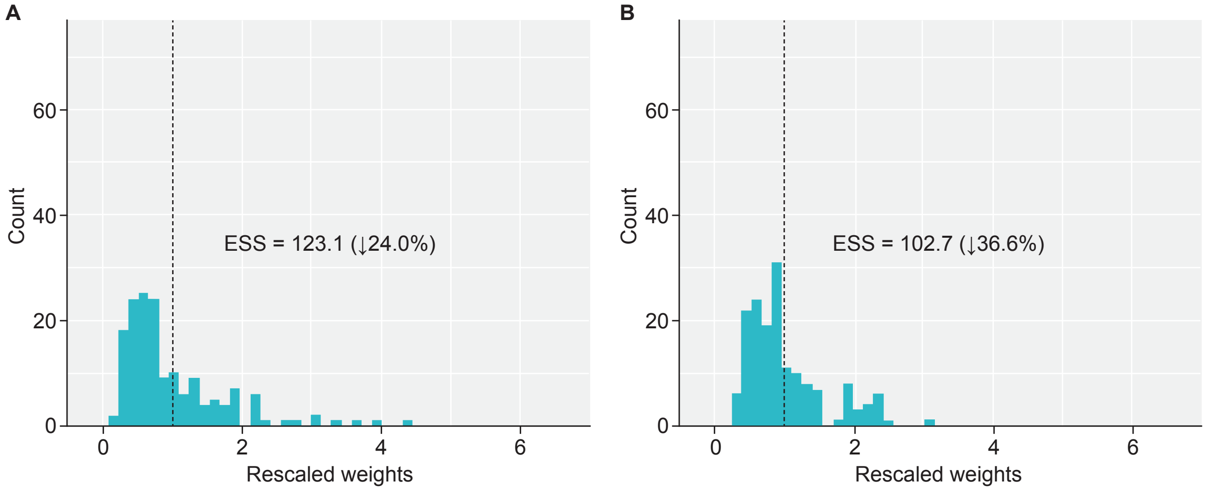

Supplementary Figure 3. **Histogram plot of weights in the primary safety analysis for (A) acalabrutinib plus obinutuzumab and (B) acalabrutinib monotherapy.**

This histogram of the rescaled weights from this MAIC analysis shows that the distribution is mainly concentrated about 1 and there are no extreme weights (>10), which would indicate that a small minority of patients had a disproportionately large influence on outcomes. The reduction in ESS (which in itself is a derivative of the raw weights) was also comparatively small (24.0% for acalabrutinib plus obinutuzumab and 36.6% for acalabrutinib monotherapy). This is an indicator of the degree of overlap in baseline characteristics across the two studies. A large reduction in ESS (>70%) has been shown to increase the level of bias in any indirect treatment comparisons.
ESS, effective sample size; MAIC, matching-adjusted indirect comparison.

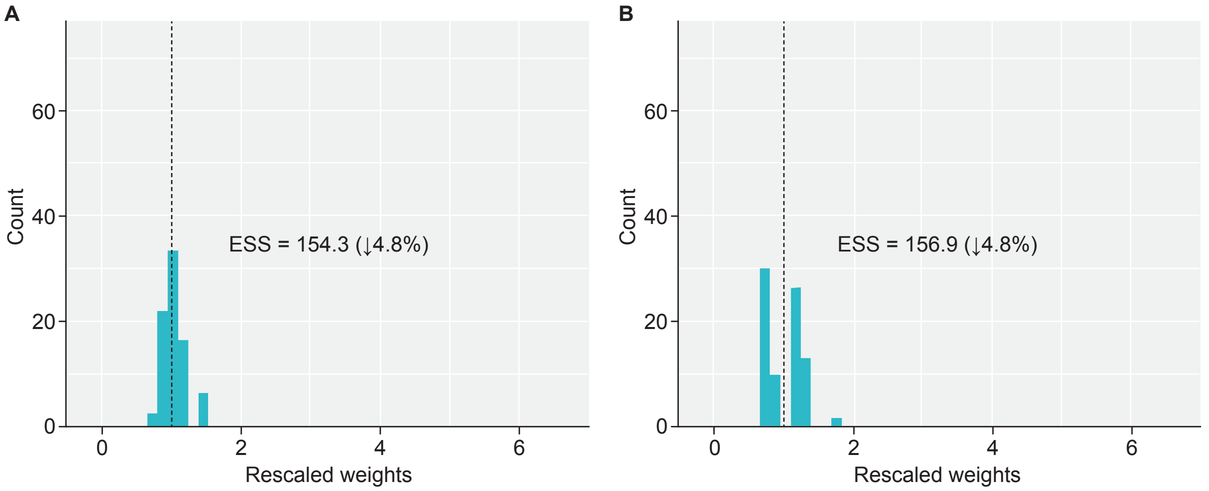

Supplementary Figure 4. **Histogram plot of weights in the safety sensitivity analysis for (A) acalabrutinib plus obinutuzumab and (B) acalabrutinib monotherapy.**

This histogram of the rescaled weights from this MAIC analysis shows that the distribution is mainly concentrated about 1 and there are no extreme weights (>10), which would indicate that a small minority of patients had a disproportionately large influence on outcomes. The reduction in ESS (which in itself is a derivative of the raw weights) was also comparatively small (4.8% for acalabrutinib plus obinutuzumab and 4.8% for acalabrutinib monotherapy). This is an indicator of the degree of overlap in baseline characteristics across the two studies. A large reduction in ESS (>70%) has been shown to increase the level of bias in any indirect treatment comparisons.
ESS, effective sample size; MAIC, matching-adjusted indirect comparison.

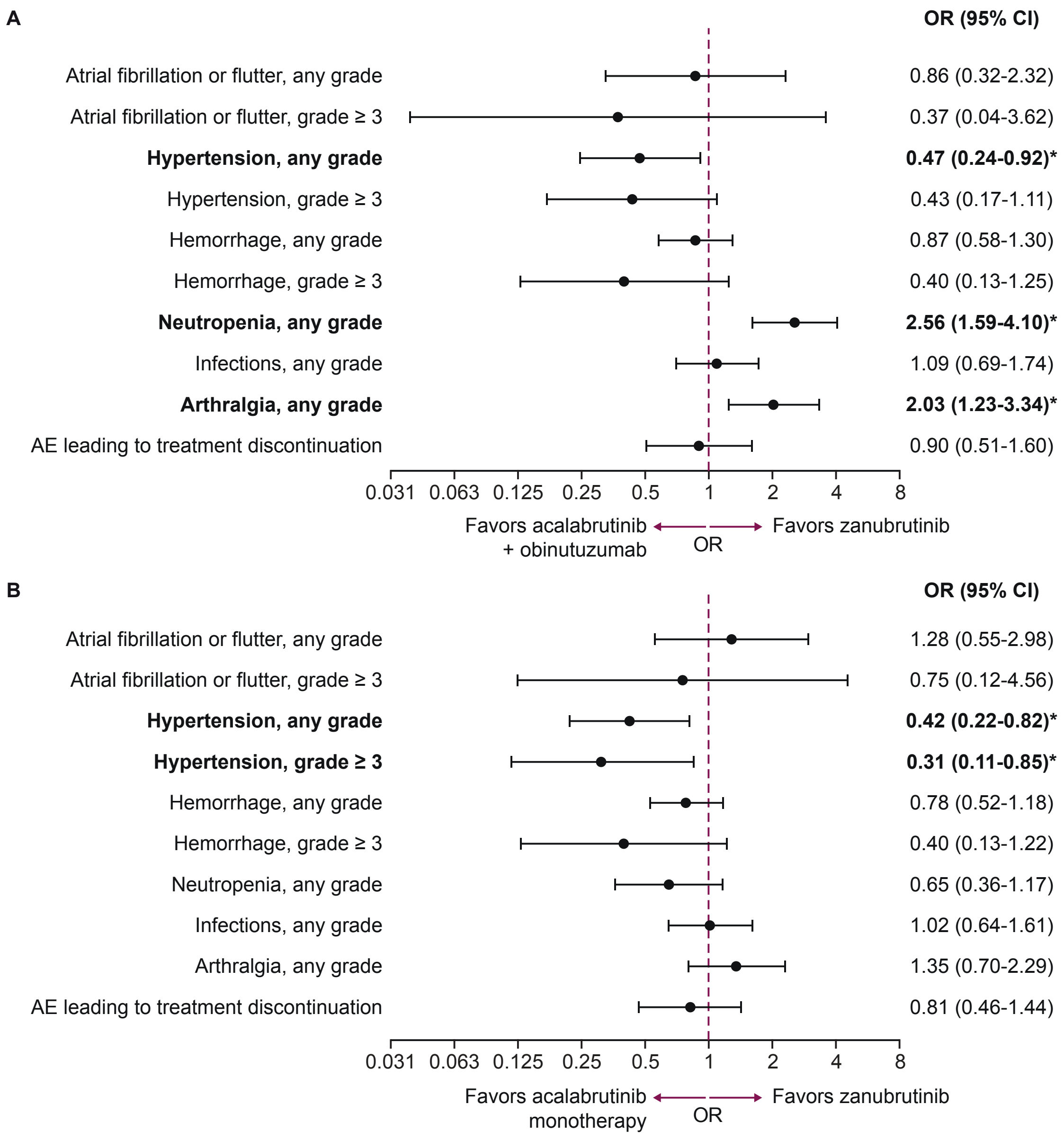

Supplementary Figure 5. Forest plot showing the odds ratio of AEs post-matching with (A) acalabrutinib plus obinutuzumab and (B) acalabrutinib monotherapy versus zanubrutinib in the sensitivity analysis that matched only on variables thought to impact safety outcomes.

Note: ORs in bold with an asterisk are statistically significant.
AE, adverse event; CI, confidence interval; OR, odds ratio.
